# Mechanisms of change and participant outcomes in a Recovery Education Centre for individuals transitioning from homelessness: a qualitative evaluation

**DOI:** 10.1186/s12889-020-08614-8

**Published:** 2020-04-15

**Authors:** Nadine Reid, Bushra Khan, Sophie Soklaridis, Nicole Kozloff, Rebecca Brown, Vicky Stergiopoulos

**Affiliations:** 1 Centre for Addiction and Mental Health, 1001 Queen Street West, Toronto, Ontario M6J 1H4 Canada; 2 Centre for Addiction and Mental Health, Department of Psychiatry, University of Toronto, 1 King’s College Circle, Toronto, Ontario M5S 1A8 Canada; 3 Centre for Addiction and Mental Health, Department of Psychiatry and Department of Family and Community Medicine, University of Toronto, 1001 Queen Street West, Toronto, Ontario M6J 1H4 Canada; 4 Centre for Addiction and Mental Health; Department of Psychiatry and the Institute of Health Policy, Management and Evaluation, University of Toronto, 1001 Queen Street West, Toronto, Ontario M6J 1H4 Canada; 5 Centre for Urban Health Solutions, St. Michael’s Hospital, Unity Health Toronto, 30 Bond Street, Toronto, Ontario M5B 1W8 Canada; 6 Centre for Addiction and Mental Health, Department of Psychiatry, University of Toronto, 100 Stokes Street, Room 6215, Toronto, Ontario M6J 1H4 Canada

**Keywords:** Recovery education, Recovery college, Homelessness, Mechanisms, Outcomes

## Abstract

**Background:**

Recovery Education Centres (RECs) are increasingly implemented to support the process of recovery for individuals experiencing mental health challenges. However, the evidence on key REC mechanisms and outcomes, particularly for diverse subpopulations or service delivery contexts is scant. This study identified mechanisms and outcomes of an REC focused on adults with mental health challenges transitioning from homelessness.

**Methods:**

Qualitative methods were used to explore in-depth the experiences of homeless and unstably housed participants experiencing mental health challenges in Toronto, Canada. Twenty service users participated in semi-structured interviews between July 2017 and June 2018, six to 14 months following REC enrollment. A realist informed interview guide explored participants’ perspectives on key REC mechanisms and outcomes. Interviews were audio-recorded, transcribed verbatim and analyzed using inductive thematic analysis. Investigator triangulation and member checking processes enhanced analytical rigour.

**Results:**

Participants perceived that program participation supported the process of recovery through several mechanisms: a judgment-free environment; supportive relationships, mutuality and role modelling; deconstruction of self-stigma; and reclaiming of one’s power. Participants described several outcomes at the personal, interpersonal and social levels, including improvements in health and well-being; self-esteem, confidence and identity; sense of empowerment, control and personal responsibility; as well as improvements in interpersonal skills, pro-social behaviours and ability to self-advocate; and increased goal development and future orientation.

**Conclusions:**

Findings suggest RECs can support the process of recovery among people transitioning from homelessness and can successfully support subpopulations experiencing mental health challenges and social disadvantage.

## Background

Recovery Education Centres (RECs) are increasingly implemented to support the process of recovery for people experiencing mental health problems and illnesses [30]. First developed in the United States (US) in the 1990s, RECs have since proliferated, and now exist in over 20 countries [31].

In complement to traditional health services, RECs use emancipatory adult education to enable participants’ recovery and pursuit of meaningful goals [12]. Principles of emancipatory adult education suggest that teaching and learning play key roles in making positive social and political reforms and creating a just and democratic society [14], partly by empowering individuals to redress undesirable or unfair situations [21]. In the context of supporting recovery among people with mental health problems and illnesses, RECs put these principles into practice by focusing on individual strengths, personal growth, and self-determination [10, 12]. The aims of RECs are to support the life goals of participants in the areas of health and well-being, education, employment, and wellness management [4, 12, 15, 27, 30]. Key characteristics of RECs include peer and professional involvement in the design and delivery of services through the process of co-production, a community focus, and inclusivity [17, 19, 29].

Given their recent proliferation, policy relevance, and potential impact, there has been considerable international interest in RECs [19]. However, despite widespread dissemination, evidence regarding how RECs support the process of recovery and participant outcomes is limited [31]. Recent qualitative research has suggested four potential mechanisms of action, including an empowering environment, relationship-building, the facilitation of personal growth, and deconstruction of traditional power hierarchies through co-production [28, 29]. Additional qualitative studies have contributed to our understanding of participant outcomes, describing improved well-being and quality of life; increased feelings of hopefulness, empowerment and belonging; and improved knowledge about and management of mental health challenges [3, 20, 28, 32].

In keeping with these findings, previous non-experimental quantitative studies of RECs in the UK have suggested improvements in several participant outcomes, including decreases in hospital inpatient days and admissions [1]. In the US, limited but similar findings have been reported. For example, a rigorous quasi-experimental evaluation of an REC in Boston found a significant short-term impact on participants’ feelings of empowerment, self-efficacy, support and affirmation [9]; and in a multi-methods evaluation of a REC in New York, participants reported improved educational and functional skills, in addition to experiencing a strong sense of community [30].

Despite the growing literature, little is known about how REC participation and outcomes might differ in diverse service delivery contexts and for diverse subpopulations, such as people experiencing homelessness and unique barriers to recovery. As homeless individuals experience complex health, mental health and social needs, recovery from homelessness has been conceptualized as largely overlapping with the process of recovery from mental health and substance use challenges in prior research [16, 18, 22, 23]. Prior studies have also highlighted the central roles of housing and choice in services, as well as the importance of social relationships, meaningful activities and valued social roles in facilitating the process of recovery for this population [16, 18, 22, 23].

The aim of this paper is to explore the mechanisms and outcomes of a Canadian REC for individuals transitioning from homelessness. We used qualitative methods to explore how REC participation supports the process of recovery in this population. As individuals facing homelessness and mental health challenges experience multiple health, socioeconomic and systemic barriers that hinder the process of recovery [24], this study can contribute to our understanding of interventions to support recovery in this disadvantaged population.

## Methods

### Intervention description

The Supporting Transitions and Recovery (STAR) Learning Centre at St. Michael’s Hospital in Toronto, Ontario was developed in 2014 as the first REC in Canada and one of the few worldwide focused on people experiencing or transitioning out of homelessness [6, 10]. Classes and workshops at STAR are offered by peers with lived experience of mental health challenges and homelessness, social and health service providers, and invited topic experts. The curriculum, including over 16 h of classes weekly, is co-designed with participants and includes topics on health and wellness, vocational skills, leadership and community engagement, hobbies and interests, interpersonal skills, conflict resolution, recovery, and life skills related to transitions from homelessness to housing such as living on a budget and working with landlords [6]. To facilitate community integration, the program uses a hub-and-spoke approach, which arranges services in a network that includes a primary location (hub) as well as secondary locations (spokes) such as the local public library, a primary care clinic for people with experiences of homelessness and employment centres [11].

STAR participants have histories of housing instability and are referred from hospitals, primary care clinics, as well as local shelters, community organizations, and other settings serving this population, or are self-referred. Participants select from a variety of courses described in a monthly catalogue, and work towards individualized learning plans [6].

### Study participants and recruitment

This qualitative study was part of a larger, mixed methods evaluation [10]. Eligibility criteria included age 16 years or older; current or recent (past 2 years) experience of homelessness or precarious housing (see Durbin et al., [10], for definitions); and capacity to consent to research participation. Purposive sampling was used to select those participants from the larger sample (*N* = 92) who were able to reflect on and provide insight into their experiences with services. Twenty-four participants were identified by the research team. Of those, two participants refused participation and one participant moved away. Recruitment continued until saturation was reached (*N* = 20) [25]. Research Ethics Board approval was obtained from St. Michael’s Hospital and the Centre for Addiction and Mental Health in Toronto, Ontario. All participants provided written informed consent.

### Data collection

In-person, semi-structured qualitative interviews with 20 participants were conducted by research staff experienced in engaging this population. Interviews took place between July 2017 and June 2018, six to 14 months following REC enrollment, to allow for rich descriptions of experiences as well as outcomes of participation. The interview guide used a realist framework to elicit rich descriptions of participant experiences, and develop a nuanced understanding of the program and its context, as well as the links between program ingredients, mechanisms and outcomes [26]. Questions were open-ended and included probes exploring motivation for enrolling in the REC, changes in the participant and the participant’s life, and how program elements facilitated recovery. Participants were also asked to reflect on any missing program elements and for suggested changes to the program. Interviews lasted an average of 65 min (range: 35 to 115 min). All interviews were audio-recorded and transcribed verbatim. Study participants were provided with a $30 honorarium and public transit fare for their participation.

### Data analysis

Inductive thematic analysis was used to analyze interview data and identify emerging themes [2], which were further refined and organized using a realist framework to identify themes relating to key mechanisms of change and participant outcomes [26]. Data was coded for positive, neutral and negative comments within themes. Investigator triangulation was used to ensure analytical rigour and trustworthiness of the data. Five participant interviews were used to develop the codebook and were later recoded using the finalized codebook [8]. Three researchers with expertise in thematic analysis independently coded three transcripts using an inductive, line-by-line approach before meeting on three occasions to compare findings, resolve differences and achieve consensus [25]. Three additional transcripts were then double-coded to establish inter-rater reliability (k = 0.72) [10]. The remaining transcripts were then coded by the same three researchers. The coding structure and relationships between themes were further revised through discussions with the study team and Principal Investigator. QSR International NVivo 9 software was used to manage and code all data. To validate accuracy and completeness of emerging themes, a member checking process was performed with three participants by a member of the research team.

## Results

The number of hours of program participation ranged from four to 249 h (mean: 80 h). Study participants were predominantly Caucasian, female, with a mean age of 44.6 years. Participants varied significantly in their level of education, from not completing high school to completing graduate school, with the majority having completed post-secondary education (*n* = 11, or 55%). All participants had been homeless in the past two years, and three were currently homeless. All but two participants were unemployed at the time of registration, with one being retired and another working part time to supplement their disability income. Key demographic characteristics of the sample are presented in Table 1.

**Table 1:** Demographic characteristics of REC participants (*N* = 20)

Characteristic		Value
Number of hours of program participation (mean (SD); median (IQR))		80.4 (50.8); 66.8; (73.1)
Gender (n (%))	Male	7 (35%)
	Female	13 (65%)
Age at time of interview in years (mean (SD); median (IQR))		44.6 (12.5); 49.0 (19.0)
Education level (n (%))	High school or less	5 (25%)
	Some post-secondary, including university, business, trade or technical school	4 (20%)
	Completed post-secondary, including university, business, trade or technical school	11 (55%)
Ethnicity (n (%))	Caucasian	16 (80%)
	Non-Caucasian	4 (20%)

Participant narratives exposed four mechanisms supporting the process of recovery and highlighted several positive participant outcomes. Participants perceived that the process of recovery was facilitated by: a judgment-free environment; supportive relationships, mutuality and role modelling; the deconstruction of self-stigma; and the reclaiming of one’s power.

Participants described several positive outcomes, within three main themes. Personal outcomes included improvements in the individual’s mental and physical health; their self-esteem, confidence and identity; as well as sense of empowerment, control, and personal responsibility. Interpersonal-level outcomes included improved interpersonal skills, pro-social behaviours and ability to self-advocate. Socially, participants described increased goal development and future orientation. Both mechanisms of change and outcomes of participation are described in detail below. Of note, although participants were asked about missing program elements and suggestions for improvements, negative experiences were not cited consistently enough to support a theme.

### Mechanisms of change

#### A judgement free zone

The majority of study participants (*n* = 14) described the REC as a space where they felt comfortable and supported to engage on their terms, in contrast to other experiences in health care settings. Participants particularly valued feeling accepted and experiencing a lack of judgment while at the program. For example, participants explained that within the program, “you’re more free to be yourself and to say what you need to say” (P114); they felt “a lot more comfortable to address my journey in recovery… through not being judgmental …” (P78) and had

a lot of trust because you’re with other people who have been in similar lived experience situations. A lot of the stress is removed of going in and starting something new. A lot of the intimidation is removed … you learn and grow together in that vital stage of starting out. Those things are very helpful. (P88)

Participants referred often to the positive environment within the REC and how it encouraged their engagement and active participation. For example, participants described “look [ing] forward to classes” and the program as “very empowering, supportive” (P78) with a “healthy learning environment that I really thrive in … it feels very positive, encouraging, and … like a safe space” (P109), one in which “everybody pretty much feels comfortable.” (P63). The nature of the program made participants feel comfortable enough to partake: “I participate more now. Very...very much. Even in stuff I thought, ‘I’m never going to be able to make it,’ I do. I try...because I’m not judged…because I’m not told, ‘Well, you can’t’” (P108).

#### Supportive relationships, mutuality and role modelling

Nearly all participants (*n* = 18) discussed the importance of social support and decreased isolation, and a sense of mutuality, reciprocity and community in the program. Participants valued the opportunity to form supportive relationships with others. They felt “it’s the interaction [I valued most] … I don’t really have many friends … so it’s a new step for me to get out there and be involved with people that are likeminded” (P63). As one participant further explained, “Just the support, the social inclusion of the atmosphere … the social network opportunities with co-members, in sharing information, in the community, resourcing with each other, that in itself is also extremely invaluable” (P78). As another participant described,

I think certainly my social group is much broader than it used to be … And that’s really what it helped, [the feeling that] I’m not alone. I’ve got this group … I don’t think I would have ended up in such a bad place if I’d had that team together this time last year. (P3)

Participants also spoke to the quality of the relationships and support, and the resulting sense of connectedness and belonging these engendered. Multiple participants described their relationships with program staff and fellow members as familial. For example, one participant described how they “feel real supported. I feel very cared about there … I feel like family, which is a nice feeling, you know?” (P160). This was especially valued by participants who did not have strong relationships with their own families:

I felt really comfortable there, I felt really welcomed. I have a new family there, I do. They’re all family members to me right now … These are my new brothers from different mothers, that’s what I call them … People I can turn to if I’m having a crisis … they’re always there … what real families are all about. (P108)

Role modelling by program clinicians, peers, staff and volunteers with relatable lived experiences was also frequently mentioned by participants. For example, participants appreciated that some of the staff and volunteers were previously program participants themselves. Participants strongly valued “hearing and seeing a good example of where I could be” (P109) and based on their observations of others’ successes, described feeling inspired and more hopeful about their own potential. As one participant explained, “looking at them … is like, ‘This could be you’” (P4). Other participants echoed this sentiment, describing it as “incredible, knowing that people out there have gone through some of the same things I’ve gone through” (P49) and how “knowing that they’ve achieved what they achieved is like a light at the end of a tunnel” (P114).

#### Deconstruction of self-stigma

The majority of participants (*n* = 15) described an ongoing change process in which they were able to actively deconstruct self-stigma though program participation. One participant described realizing “that how I see myself and how other people see me is quite different” (P1) and many spoke specifically about learning to reframe previously negative perceptions of themselves and their experiences. One participant described learning to think about her lived experience less negatively because the program “show [ed] that there are outlets for that [lived experience] where you can see … that perhaps your lived experience, which might have seemed like just a bad time or a mess, can be … something productive for yourself and other people” (P88).

Other participants described the process of overcoming feelings of shame. For example, one participant spoke about how their lack of education made them feel “below everybody,” but that the program helped them feel less ashamed of not having had any “education or training. I’ve never been ready to go to school … but they’re like, ‘It’s okay to be where you’re at’” (P160); and another described learning to overcome shame related to their substance use:

When you feel really shitty about yourself and you’re like, ‘Oh, I’m a bad person because I smoked meth,’ … and you look at them [someone with similar experience] and you think, ‘I don’t think this person is a bad person. Why don’t they think they’re a bad person because they did the same thing?’...It’s relatable. It makes me think a lot” (P109).

#### Reclaiming one’s power, being in charge of one’s recovery

Half of the participants (*n* = 10) described realizing, though participation, that they were in charge of their own learning and recovery. This related to the program’s emphasis on participatory processes and self-determination, giving participants a sense of power and control. Some participants described “learning to get up at a certain time to get out the door” (P160) and learning “to go in and actually make an effort to talk about my learning … so there’s that responsibility-building component” (P3). Participants appreciated learning they “get to direct where I’m going and what I’m doing and what I want to do” (P62) and that as a result, they began to feel “validated and that you have a say in things and you *can* make a change in what’s going on” (P88).

Being in control allowed participants to focus on gaining knowledge and on building competencies and skills in areas of importance to them. For example, a participant focused on improving their health described valuing “learning how to self-care, learning how to identify what’s wrong, learning how to communicate, all of those skills I’ve been in very short supply of” (P1) and another described more generally “learning how to re-wire your brain … learning new behaviours as well as using those behaviours right away to create an action plan” (P88). Others who were particularly focused on gaining employment described pursing education and skills training that would help them to move forward in that respect. For example, one participant described how

Through [the program], I registered with [a training centre] as an independent student. I’ve gotten courses that can very much complement my CV: non-violent crisis intervention, the CPI [Crisis Prevention Institute] first aid, naloxone training. All of these very real, functional, real world applied skills. (P78)

### Participant outcomes

#### Personal outcomes

Participants described positive outcomes in several areas, including *heath* and *well –being; self-esteem, confidence* and/or *sense of identity*; and in *feelings of empowerment, control* and *personal responsibility*.

##### Health and well-being

Participants reported improvements in both their health and their ability to manage their health and well-being (*n* = 13). As one participant summarized, “I’m a lot healthier, physically, emotionally. I would say I’m a healthier me” (P136), while others commented “not being super depressed … I got sober in November … I don’t think I would have done it without [the program]” (P109) and getting “physically health, physically fit. Extremely fit. Mentally, as well..” (P115).

Multiple participants also described being better able to manage or cope with their mental health challenges as a result of program participation. As one participant described:

I feel that through my experience there [at the program], I’m able to cope with situations better than I would have before … it really provided me with the tools to deal … ‘Managing Symptoms’ was probably the one [course] that was most useful because I hadn’t really recognized the symptoms before. So now I can identify if my mood is changing or if my behaviour is changing or if my activity is changing, which are indicators that something’s not right. (P3)

Other participants explained that they gained “different ways of trying to manage my symptoms now” (P1); now “know [ing] enough about how to cope” (P160); and how “just the support, the social inclusion of the atmosphere, that has greatly improved my ability to manage my mood, depression, anxiety” (P78).

##### Self-esteem, confidence, sense of identity

Following from the processes of deconstructing self-stigma, along with knowledge, competency- and skill-building, most program participants (*n* = 15) described significant improvements in their *self-esteem, confidence* and/or *sense of identity*.

Participants explained that the program “built my confidence” (P115) and made them “feel like I can accomplish, I don’t know what, just accomplish … I kind of learned that I had some value, going to [the program], and then that value has or is slowly going outward” (P133). As a result of program participation, participants described reconstructing a more positive sense of identity and self-worth. For example, one participant explained that “In terms of being marginalized by my lived experience as this vulnerable, victimized person, I don’t feel so much that way anymore” (P88); and another described: “I see myself in a better light. Way better light. I think I hated myself, and I had really shitty self-esteem … and now I feel a lot better about myself … improved self-esteem for sure” (P109). Another participant echoed, “I see myself as a person of value now versus another nobody” (P160); and yet another explained:

[The program has] gotten my self-esteem right back up there … It’s changed me totally … I used to hate myself ... and now I’m like, ‘No, I’m not worthless, I’m an individual.’ And I’ve learned that from them. (P108)

##### Empowerment, control and personal responsibility

Alongside increased self-esteem and confidence, in the context of reclaiming one’s power and building competencies and skills, participants reported increased feelings of *empowerment, control* and *personal responsibility* in other aspects of their lives (*n* = 11). For example, one participant, when asked about the most significant change experienced, replied:

Biggest change is becoming more responsible … I definitely feel more responsible … I definitely feel more empowered … I feel now that I have been empowered enough to speak to others about what I’m going through and see how people can help in that. (P114)

Multiple participants described feeling “way more in control” (P4):

I have much more control than when I started … It’s given me the tools to help me control aspects of my life that I didn’t feel as though I had control over … in terms of being able to make decisions, to take action on things that needed to be done, how to build that support system so that when I feel as though I lose control, [I can] get back in control … I’m more optimistic that that [traumatic] event will not control me, I can control it. (P3)

Participants also described an increased sense of responsibility and how the program “actually help [ed] me do more things for myself” (P63). For example, one participant described how they became “more responsible and accountable for myself again” (P160); and another explained how the program

has helped me build up confidence in terms of committing to something again. I used to be notorious for cancelling my doctors’ appointments and sometimes I wouldn’t even call …. Has it made me a better housekeeper? A little bit … but I have also taken responsibility for a lot of other things in terms of making appointments and doing things. (P1)

#### Interpersonal outcomes

Following from their experiences in a safe, supportive and enabling environment, nearly two thirds of participants (*n* = 13) described significant improvements in their *interpersonal skills,* and in their *desire and ability to self-advocate* and *enact pro-social behaviours*. Many participants spoke about how participation helped improve their communication skills. As one participant described:

… [the program] has shown me how to put my idea across without offending the person. ...Just taught me to be more mindful, and how to read body language. They say 90% of communication is done non-verbally … Without taking these classes, I don’t think I would have gained that skill. (P68)

As a result of improved communication skills, several participants described how they “began to open up more” (P88) and became “certainly much more open and willing to discuss how I feel and how I react … and I would never have done that before” (P3).

Relatedly, participants reported improvements in their self-advocacy. Numerous participants described being better able to “stand my ground” (P50); be “more outspoken. I stand up for myself more. I promote myself more and I’m not afraid to say what is” (P62); and “articulate, advocate for myself stronger than I normally would. To be a little more tenacious” (P78). As another participant explained,

Now I’m fighting for my rights … it got me what I needed … I wouldn’t have made noise before I started at [the program]. It’s awesome … they really taught me how to be positive, how to be out there, just to be out there and let my voice be heard … I’m able to voice what I need. (P108)

Participants also described improvements in pro-social behaviours. For example, one participant described how they no longer “start [verbal] fights ... I don’t have outbursts” (P160). For some, this was the direct result of the positive environment and role modelling that had occurred. One participant described how seeing others act positively toward each other helped him adopt similar behaviours:

They [peers] appear to be very concerned with how everybody’s doing, and it’s interesting because that is an attribute which rubs off. I had experienced in classes people getting upset and storming out or blowing up or whatever, and being truly concerned about their well-being. And it’s not always the way I was … I’m happy about it. (P62)

#### Social outcomes

Socially, program participants described increased *future orientation and goal development* in support of their recovery. Over half of participants (*n* = 12) reported having clearly developed goals and a positive outlook following program enrollment. For some participants, future goals pertained to maintaining their health and well-being, with several specifically noting a goal of staying sober. Participants described goals like having “a healthy, satisfying life … and to find that balance that I didn’t have before” (P3) and to “continue with fitness … a big goal is to get off [provincial disability allowance] …” (P115).

Other participants had developed education- or career-related goals as a result of program participation. One participant described how attending classes helped them realize they could attend classes anywhere: “... helped me understand certain things about myself … it helped me go back to work … and dealing with another class setting set me up to actually go for a bigger class, like maybe [a local college]” (P4). Several participants specifically described the goal of becoming peer support workers. For example, one participant described aiming to “get as much training as I can right now to put towards my career goals … I’m really geared towards peer support training” (P63).

Finally, participants described goals of finding meaning in their lives, often by giving back to the community in some way. For example, one participant explained that “the big goal is finding meaningful work or volunteer thing … that’s a big priority for me now … so I have that goal to find meaningful work or a meaningful way to use my time” (P115). Others echoed this desire to “be involved with life. I need to have a purpose” (P136) and “help other people the way I’ve been helped” (P160). As one participant summarized the impact of the program on their sense of future-directedness:

I’ve started thinking more about my future and the direction I want to take it and that makes me really happy because I don’t think I was thinking about my future for a really long time. I think I was just trying to really struggle to get through every day. And now I’m in a place where every night I can plan my next day … every day I can think about where I want to go, and what I want to do, and whether this is contributing towards this beautiful future I have. (P109)

## Discussion

This is the first study to examine the mechanisms and outcomes of a Recovery Education Centre for individuals transitioning out of homelessness, a population with complex support needs that is traditionally difficult to engage in services.

Our findings largely support and are supported by previous research [28] and extend findings to a disadvantaged subpopulation with unique barriers to recovery. Similar to Toney et al. [28], our findings highlight the essential role of an enabling environment; the importance of relationships between staff, volunteers and participants in giving and receiving social support, and to experience mutuality; the value of self-directed learning and opportunity to build skills and competencies in personally meaningful areas; and the overarching personal growth process that occurs among participants, particularly in terms of rebuilding a more positive sense of self. Notably, our findings are unique in identifying role modelling and the deconstruction of self-stigma as key program mechanisms supporting the process of recovery in this population.

Our findings also suggest that program mechanisms interact to reinforce each other, consistent with the supposition that the overarching mechanism underlying recovery education is not simply knowledge acquisition but a more active, individual and transformative process [19, 29]. For example, in our study, role modelling by peers with lived experience contributed to the deconstruction of self-stigma by contradicting previously internalized negative stereotypes about individuals experiencing homelessness and mental health or addiction challenges and their potential for recovery, which in turn supported the development of more positive self-identity and increased feelings of self-worth. This finding is consistent with previous literature suggesting self-stigma is a barrier to recovery [7] and is the first evidence to support the hypothesis that RECs help to dismantle self-stigma by introducing participants to peers who are leading and succeeding [19]. Similarly, the process of reclaiming one’s power and gaining personally meaningful knowledge and skills contributed toward increasing sense of self-efficacy and empowerment. This particular set of interactions is consistent with a well-known model of the empowerment process [5] and aligns with previous evidence suggesting RECs are associated with increased empowerment among participants [19]. By supporting existing evidence and proposing additional program mechanisms, our findings help to further understand the theory of change underlying REC outcomes such as improvements in self-esteem, empowerment, health and well-being.

Our findings are also consistent with and expand upon previous studies describing outcomes among REC participants, including improved self-understanding and confidence [28, 32]; empowerment, self-efficacy and support [3, 9]; hopefulness [3, 20]; and a sense of community [30]. In addition to these outcomes, our findings suggest participants transitioning out of homelessness specifically develop improved interpersonal skills and prosocial behaviours, experience improvements in health and well-being, and become more goal- and future-oriented as a result of REC participation. While prior studies have documented evidence of decreased service use among REC participants, our interview guide did not specifically probe discussion of this outcome, nor did this theme emerge spontaneously from our sample. Taken together, our findings support prior research by Toney et al.’ [28] and the commonly employed CHIME recovery outcome framework [17], which proposes that connectedness, hopefulness, identity, meaning and empowerment are key recovery outcomes.

Our findings suggest that similar mechanisms of change and participant outcomes are observed in a subpopulation experiencing or transitioning out of homelessness as in broader samples. In this context, it is important to highlight that the key elements and mechanisms of change in RECs are aligned with the central roles of choice, social relationships, meaningful activities and valued social roles, previously identified as central in facilitating the process of recovery for this population [16, 18, 22, 23], and in keeping with this populations’ service needs and preferences [13]. This, in turn, suggests that RECs can have an important role to play in supporting the process of recovery within the homeless services sector.

Our study has several strengths including the rigorous qualitative design, the successful engagement of a participant population that is historically challenging to engage, and the generation of rich descriptions of both program mechanisms and outcomes. Member checking strongly validated emerging themes. Our results are likely to be generalizable to other RECs given the close fidelity of our program to the REC model [10, 29], suggesting that our findings can be used to inform program and policy development in similar contexts and with similarly marginalized populations.

Study limitations include the cross-sectional design and a study sample with a somewhat higher education level than seen in the general homeless population. It is possible that participants with different demographic backgrounds or those less readily able to express themselves may experience different mechanisms and outcomes than those described in the current sample. In addition, given the responsive nature of the program, it is possible that interviews conducted at a later date in program participation may elicit additional mechanisms and outcomes.

This study adds to a growing literature on key REC mechanisms and outcomes, an area in need of further development. Future research should seek to further explore mechanisms of change and health and social outcomes for diverse populations facing multiple barriers to personal recovery. Future research should also attend to potentially negative experiences, early disengagement from RECs, and unintended consequences of participation, to help guide further improvements in the field.

## Conclusion

Among individuals experiencing or transitioning out of homelessness, RECs may successfully engender key recovery mechanisms and outcomes and complement traditional approaches to supporting this marginalized population. Study findings add to a growing evidence base on the role of RECs in supporting the process of recovery among individuals experiencing mental health challenges.

## Data Availability

The datasets used and/or analysed during the current study are available from the corresponding author on reasonable request.

## Abbreviations

REC: Recovery Education Centre
STAR: Supporting Transitions and Recovery
